# The second victim phenomenon in medical education: Development of learning objectives based on the National Competency-based Catalogue of Learning Objectives for Undergraduate Medical Education (NKLM) 

**DOI:** 10.3205/zma001786

**Published:** 2025-11-17

**Authors:** Tobias Bexten, Jens Christian Kubitz, Anne Kamphausen, Reinhard Strametz

**Affiliations:** 1 Helios Dr. Horst Schmidt Clinic Wiesbaden, Clinic for Interdisciplinary Intensive Care Medicine and Intermediate Care, Wiesbaden, Germany; 2 Teaching Hospital of the University Medical Centre Mainz, Mainz, Germany; 3 Paracelsus Medical University, Nuremberg Hospital, Clinic for Anaesthesiology and Operative Intensive Care Medicine, Nuremberg, Germany; 4 RheinMain University of Applied Sciences, Wiesbaden Business School, Wiesbaden Institute for Healthcare Economics and Patient Safety (WiHelP), Wiesbaden, Germany

**Keywords:** second victim phenomenon, second victim, National Competency-based Catalogue of Learning Objectives for Undergraduate Medical Education (NKLM), patient safety, peer support, learning objectives second victim

## Abstract

**Background:**

The second victim phenomenon describes the psychological, cognitive and physical reactions of healthcare professionals who are directly or indirectly involved in adverse patient events or errors and are themselves impaired as a result. Common symptoms include anxiety, guilt, grief, depression and burn-out, which can significantly restrict their ability to work. Surveys in German-speaking countries show that the prevalence of the second victim phenomenon is as high as 89% across all healthcare professions.

**Objective:**

This paper aims to synthesise learning objectives pertaining to the second victim phenomenon from the current literature and thus provide a basis for training medical students.

**Methods:**

Design: Qualitative synthesis of relevant categories using “best fit” framework synthesis based on the European Researchers’ Network Working on Second Victims. Data collection: Literature search in PubMedCentral, MEDLINE, Cochrane and CINAHL based on the categories. Data analysis: Qualitative document analysis according to Mayring with synthesis of the learning objectives and definition of the depths of competency: According to the depths of competency in knowledge, practical knowledge, and practical skills, based on the taxonomy of the National Competency-based Catalogue of Learning Objectives for Undergraduate Medical Education (NKLM).

**Results:**

The analysis resulted in the definition of a framework catalogue with four subcategories: I: Basic concepts and definition of the second victim phenomenon, II: Symptoms of the second victim phenomenon and need for support, III: Intervention options, and IV: Contextualisation of the second victim phenomenon in the broader context of employee welfare. These categories were assigned to seven areas of knowledge and, based on this, seven learning objectives with their respective depths of competence were defined.

**Conclusion:**

In this study, seven evidence-based learning objectives concerning the second victim phenomenon were defined for medical students and systematically integrated into the NKLM’s taxonomy. The results provide a structured basis for anchoring this topic into the curriculum in order to impart knowledge about the second victim phenomenon early on and minimise long-term, negative consequences for healthcare professionals.

## Zusammenfassung

**Hintergrund:**

Das Second-Victim-Phänomen beschreibt die psychischen, kognitiven und körperlichen Reaktionen von Fachkräften im Gesundheitswesen, die direkt oder indirekt an unerwünschten Patientenereignissen oder Fehlern beteiligt waren und dadurch selbst beeinträchtigt wurden. Häufige Symptome sind Angst, Schuld, Trauer, Depression und Burn-out, die die Berufsfähigkeit erheblich einschränken können. In Befragungen im deutschsprachigen Raum wurde ermittelt, dass die Prävalenz des Second-Victim-Phänomens über alle Gesundheitsberufe hinweg bis zu 89% beträgt.

**Zielsetzung:**

Ziel dieser Arbeit war es, Lernziele über das Second-Victim-Phänomen aus der aktuellen Literatur zu synthetisieren und so eine Grundlage für die diesbezügliche Ausbildung von Medizinstudent:innen zu definieren.

**Methodik:**

Design: Qualitative Synthese relevanter Kategorien nach der Methode der „best fit“-Framework-Synthese auf Basis des European Researchers’ Network Working on Second Victims. Datenerhebung: Literaturrecherche auf Grundlage der Kategorien über PubMedCentral, MEDLINE, Cochrane und CINAHL. Datenanalyse: Qualitative Dokumentenanalyse nach Mayring mit Synthese der Lernziele und Definition der Kompetenztiefe: In Anlehnung an die Taxonomie des Nationalen Kompetenzbasierten Lernzielkatalogs Medizin (NKLM), nach den Kompetenztiefen Wissen, Handlungswissen und Handlungskompetenz.

**Ergebnisse:**

Die Analyse resultierte in der Definition eines Rahmenkatalogs, der vier Teilkategorien umfasst: I: Grundbegriffe und Definition des Second-Victim-Phänomens, II: Symptome des Second-Victim-Phänomens und Bedarf an Unterstützung, III: Interventionsmöglichkeiten und IV: Kontextualisierung des Second-Victim-Phänomens im weiteren Spektrum der Mitarbeiterfürsorge. Diesen Kategorien wurden sieben Wissensbereiche zugeordnet und auf dieser Basis wurden sieben Lernziele mit den jeweiligen Kompetenztiefen definiert.

**Schlussfolgerung:**

In der vorliegenden Studie wurden sieben evidenzbasierte Lernziele zum Second-Victim-Phänomen für Medizinstudierende definiert und systematisch in die Taxonomie des NKLM integriert. Die Ergebnisse bieten eine strukturierte Grundlage für die curriculare Verankerung dieses Themas, um frühzeitig Wissen über das Second-Victim-Phänomen zu vermitteln und langfristige negative Folgen für Gesundheitsfachkräfte zu minimieren.

## 1. Background

In the early 2000s, American internist Albert Wu coined the term “second victim phenomenon” (SVP) [1], which has since been used to describe a syndrome that occurs in healthcare professionals (HCPs) after critical incidents. It manifests itself in psychological, cognitive and physical symptoms such as anxiety, guilt, grief, depression, dissatisfaction and burn-out [2], [3], [4], [5], [6]. SVP is associated with maladaptive coping mechanisms such as defensive medicine, post-traumatic stress disorder, job turnover and suicide [7], [8], [9], [10]. Although up to 89% of HCPs exhibit characteristics of a second victim (SV), the term is still relatively unknown [11], [12]. The risk of experiencing SVP is already 25% during undergraduate study [13]. Despite this, SVP has so far been inadequately addressed in medical training [14]. At the same time, factual knowledge, reasoning and decision-making skills, as well as the ability to take appropriate action in relation to SVP are essential to mitigate long-term harm to HCPs [15]. In addition to the psychological effects, the long-term consequences also include the HPC’s resultant state, which can entail positive and constructive growth as well as negative outcomes, such as dysfunctional survival in the workplace or leaving the workplace [16], [17], [18]. 

## 2. Objective

**Figure 1:**
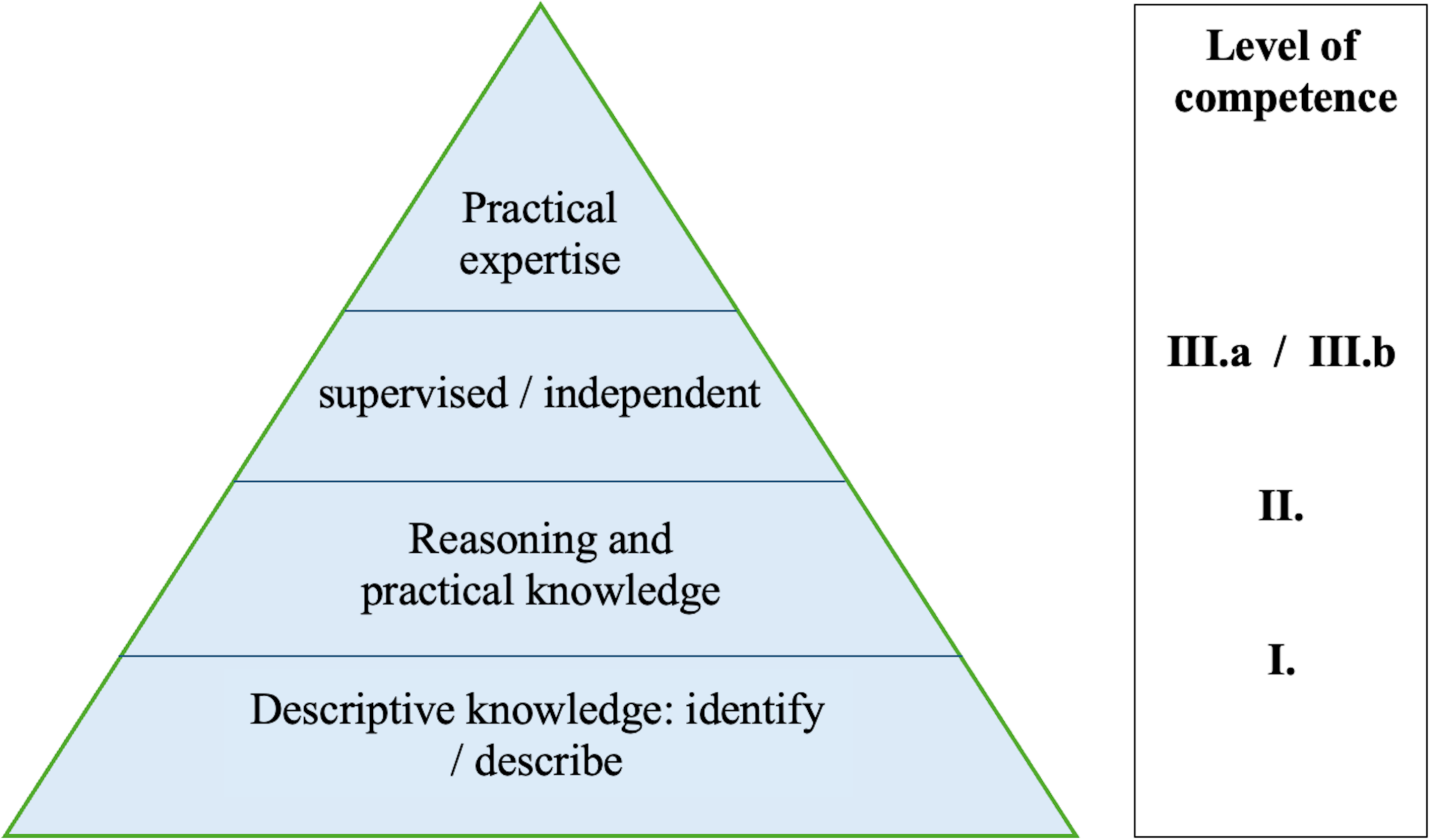
Tiered model of competence levels This illustration is based on the competence model of the National Competency-based Catalogue of Learning Objectives for Undergraduate Medical Education (NKLM), which distinguishes between descriptive knowledge (Level I) and fully fledged practical expertise (Level III.a/b).

The aim of the study was to define SVP learning objectives that every HCP should achieve. Evidence-based content was also assigned to these learning objectives. The learning objectives were classified according to the taxonomy of the National Competency-based Catalogue of Learning Objectives for Undergraduate Medical Education (NKLM) into three levels of competence (see figure 1 (Fig. 1)) and assigned to semesters [19], [20], [21], [22], [23], [24], [25]. 

## 3. Methods

### 3.1. Design

The study followed a qualitative approach (qualitative evidence synthesis) based on “best fit” framework synthesis and Mayring’s category formation [26], [27], [28]. The European Researchers' Network Working on Second Victims (ERNST) served as the starting point for developing a theoretical framework [29] (see attachment 1 , figure S2). 

### 3.2. Data collection

Data collection was carried out in two consecutive steps. First, topics relevant to SVP were synthesised. The information provided by the ERNST network was analysed, and relevant categories were developed as a theoretical framework. Subsequently, further topics, keywords and concepts were assigned to these categories based on secondary literature from ERNST. In the second step, a comprehensive literature search was conducted, primarily via PubMedCentral on 7 April 2024; this was supplemented by a second search in the MEDLINE database (via EBSCO) to identify additional indexed articles. Cochrane and CINAHL (via EBSCO) were also included in the search. Search terms derived from the previously identified categories and studies were used, particularly those that appeared repeatedly. These are summarised in attachment 1 , figure S1. Studies were included if they were directly related to SVP and had been published after the initial description by Wu in 2000 [1]. Studies not written in German or English were excluded, as were questionnaire validations, concept descriptions and redundant overviews. A flow chart showing study selection is presented in attachment 1 , figure S1. 

### 3.3. Data analysis

Data analysis was carried out in a multi-step process. First, the studies were categorised according to the previously developed framework. In the next step, the basic knowledge and learning objectives were extracted. Recurring questions and topics were identified and recorded as keywords under the individual categories. Topics that were mentioned multiple times and those that were addressed in ERNST publications were taken into account. Based on this base of knowledge, learning objectives were formulated in which the extracted content was consolidated. 

These learning objectives were then discussed and adapted by the authors.

In a third step, the learning objectives were divided into three levels of competence: factual knowledge, reasoning and decision-making skills, and practical skills. This division was based on the requirements students are expected to fulfil and the complexity of the tasks to be performed in relation to SVP, depending on the students’ current level of training. In accordance with the NKLM, chronological distinctions were made: first to fourth semesters, fifth to sixth semesters, seventh to tenth semesters, and the practical year (PY). Applying this structure enables the systematic teaching of skills to deal with SVP in a manner appropriate to the level of training. 

## 4. Results

### 4.1. Overview of current studies

The current state of research provides a good overview of SVP. In the category “symptoms and need for support”, comprehensive studies are available on different populations (doctors, nurses, students) and in different work environments (emergency rooms, intensive care units, preclinical). At the same time, the methodology is very heterogeneous (qualitative, quantitative, mixed methods), with both validated instruments and pure observations being used [11], [30]. With regard to possible interventions, there is also great heterogeneity in terms of the type of intervention and the target group [31], [32], [33]. There are a sufficient number of studies for the fourth category, “contextualisation of SVP”, some of which are theory-driven or exploratory, while others explore statistical correlations.

### 4.2. Framework

A framework consisting of four categories was defined and to which seven learning objectives have been assigned. These categories comprise: I. Basic concepts and definition of SVP, II. Symptoms of SVP and the resulting need for support, III. Intervention options, and IV. Contextualisation of SVP in the broader context of employee welfare (see table 1 (Tab. 1) and attachment 1 , figure S2). 

### 4.3. Knowledge base, learning objectives and depths of competence

In the following section, the learning objectives are presented according to categories I to IV. For each learning objective, the basic knowledge is first formulated, followed by the learning objective and the corresponding level of competence. An overview can be found in attachment 1 , table S2: Overview of learning objectives based on the NKLM taxonomy. 

#### 4.3.1. Category I: Basic concepts and definitions of SVP

##### 4.3.1.1. The second victim (SV)

*Basic knowledge, learning objective 1: *Errors, mistakes and undesirable events occur wherever people work. These can be the result of individual actions or system-related factors [34], [35]. 

Patients are among the “first victims”, while the HCP is the SV. Internationally, the term SV is defined as follows: *“Any healthcare worker, directly or indirectly involved in an unanticipated adverse patient event, unintentional healthcare error, or patient injury and who becomes victimized in the sense that they are also negatively impacted”* [36].

*Learning objective 1:* The graduate can define the term “second victim” and contextualise it using examples.

*Depth of competence: *The ability to define an SV and describe the concept in one's own words enables connections with other aspects of SVP.

##### 4.3.1.2. The second victim phenomenon (SVP)

*Learning objective 2: *SVP does not have any pathological significance in itself, but it can manifest as an illness if the psychological stress is not addressed and processed [37]. The experience of an SV is characterised by psychological and physical symptoms and thus influences, among other things, the victim’s working life. Symptoms or effects and consequences include shock, fear, guilt, shame, grief, insomnia, restlessness, depression, aggression, loneliness, loss of quality of life, palpitations, fatigue, dissatisfaction with oneself, an increase in avoidable mistakes at work and a decrease in work performance, an increased need for control and problems with work routines [30], [38], [39], [40], [41], [42], [43], [44], [45], [46], [47], [48], [49].

*Learning objective 2: *The graduate can describe the subjective experience of SVP. They have the ability to reflect on their own experience in relation to SVP and to deal with their feelings. 

*Depth of competence: *Familiarity with the subjective experience of SVP and being able to describe it in one’s own words enables mindfulness and the ability to deal with its symptoms.

#### 4.3.2. Category II: Symptoms of SVP and need for support

##### 4.3.2.1. The phases of SVP

*Learning objective 3: *The development of SVP symptoms was prototypically divided into six stages in 2007 (see table 2 (Tab. 2)). The individual stages can be experienced simultaneously or in recurring cycles [50].

1. Chaos and accident response: The HCP involved becomes aware of the adverse event and must act to stabilise the patient while they experience internal turmoil.
2. “What if ...?” This phase is characterised by self-doubt and flashbacks.
3. Awareness of one’s own role: the SV seeks help from those around them, but doubts their professional future and fears the judgement of others.
4. Uncertainty at the institutional level: questions such as “Will I keep my job?” and interactions with colleagues dominate.
5. Emotional processing: peer support from colleagues, friends and family is crucial.
6. Overcoming:

- Change of job: the SV is unable to process the situation and leaves their field of work/patient-related activities.
- Survival: the SV remains in their field of work, coping with their tasks partially or dysfunctionally.
- Personal growth: the SV processes the experience positively and learns from it [12], [18], [50], [51], [52], [53] (see table 2 (Tab. 2)).

*Learning objective 3: *The graduate can name the stages identified by Scott et al. [50] and explain the symptoms and actions based on these stages. Stages one to five can be summarised, while stage six can be specified in more detail based on the three possible outcomes. 

*Depth of competence:* Familiarity with the experience of SVP and being able to describe it in their own words enables graduates to reflect on their experiences. At the same time, it is important to describe the first five stages, bearing in mind that three different outcomes can result from them. 

##### 4.3.2.2. Prevalence, triggering events and recovery time

*Basic knowledge, learning objective 4: *SVP affects personnel in all healthcare professions, starting during their studies and training. In German-speaking countries the prevalence is between 53% and 89% [54], [55], [56]. High prevalences of up to 90% have been reported globally [57], [58], [59], [60], [61], [62], [63], [64], [65], [66], [67]. During training and studies the prevalence is between 12% and 25% [12], [67], [68], [69]. 

Yet only about 10% of doctors and 25% of nursing staff are familiar with the term SVP [11], [12].

SVP does not necessarily have to stem from harm to a patient. Rather, approximately 35% of SVs cited aggressive behaviour by patients as the decisive factor, while near misses were the cause in 12.4% of cases [11], [12], [55], [70], [71] (see table 3 (Tab. 3)).

A total of 30% of SVs recover within a week, approximately 25% within a month, and 15% within a year. Approximately 10% of SVs do not recover completely [70], [72], [73], [74].

*Learning objective 4: *The graduate can identify prevalence, triggering events and the expected recovery times for an SV.

*Depth of competence: *Knowing the triggering events and the high probability of making a full recovery from SVP is fundamental to prevention. Only an SV who can recognise themselves as such will seek help when necessary. 

#### 4.3.3. Category III: Intervention options

##### 4.3.3.1. Preventive measures 

*Basic knowledge, learning objectives 5 to 5.1.2: *The emotional and psychological consequences of SVP can lead to defensive medicine, depression, sleep disorders, PTSD, job turnover, job abandonment and suicide [6], [75], [76], [77], [78], [79], [80], [81], [82]. Defensive medicine, in particular, directly affects the patient, as it can lead to both overtreatment (unnecessary imaging, unnecessary referrals) and risk avoidance. This affects both the individual and the surrounding system and can lead to a decrease in work performance. The aim must be to recognise SVP at an early stage and disrupt negative spirals [83], [84], [85], [86]. In 2010, Scott et al. presented a three-step system to counter SVP [87]:

1. Colleagues provide the SV with a sense of stability immediately after the event and support them in the further care of the patient.
2. Professionally trained colleagues recognise signs and symptoms of SVP and offer basic personal support.
3. Psychiatric outpatient services provide professional help [87], [88], [89], [90].

Approximately 60% of SVs receive sufficient support in the first stage, whereas about 10% require the highest level of therapeutic care [57], [87], [88], [89], [90]. 80% of affected SVs would like to receive support from the team [9], [77], [87], [90], [91], [92], [93], [94], [95], [96], [97].

**Figure 2:**
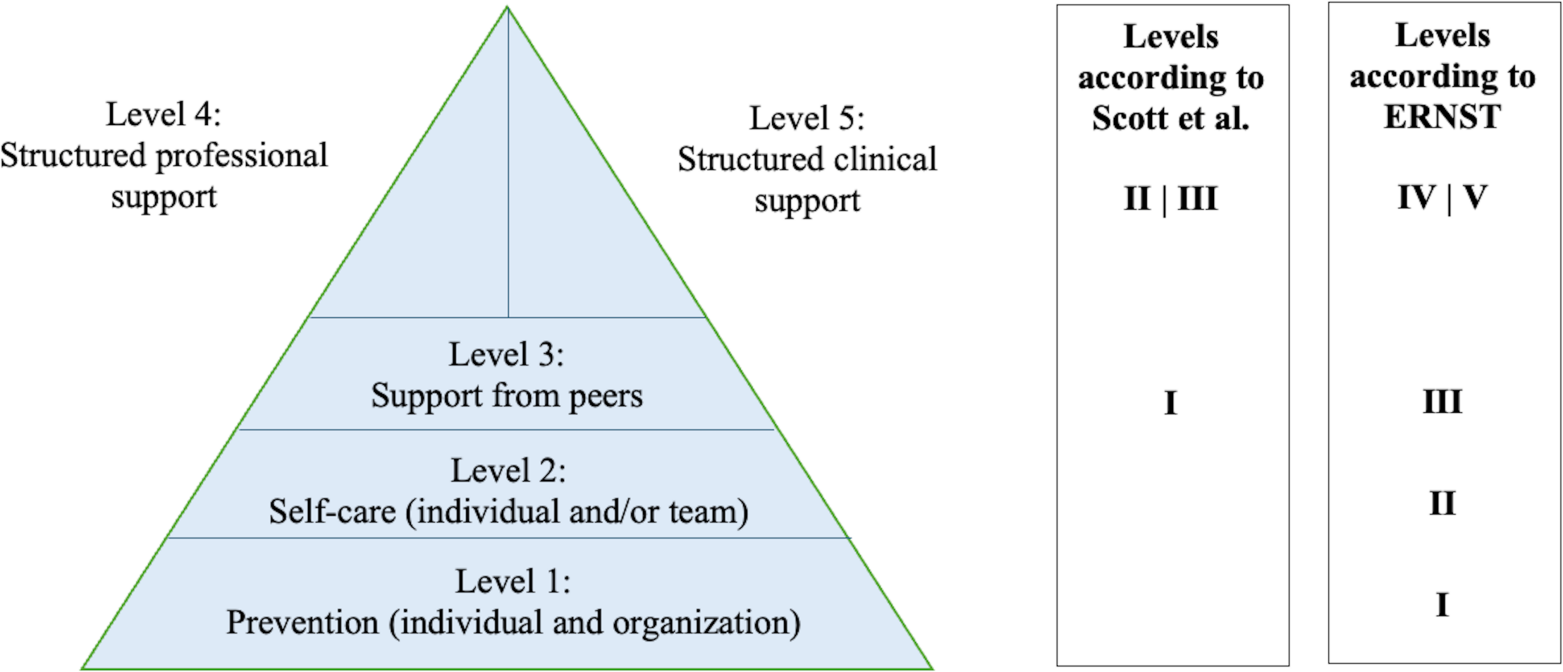
Tiered model of support The figure shows five hierarchical levels of psychosocial support, starting with preventive measures and ending with structured clinical support, and is based on the models by S. Scott et al. and the European Researchers' Network Working on Second Victims [98], [100].

In 2023 the model was revised by ERNST and now comprises five stages. While the upper three levels are consistent with the model developed by Scott et al., a foundation consisting of two levels has been added. The upper three levels come into play when a key event has occurred. The two new levels address the ability of a person or system to be prepared for an event or to respond immediately to it [98]. Examples of level one include investing in good collegial relationships, a supportive culture, a blame-free environment, a family-oriented environment, and education about SVP [80], [99], [100]. Level two includes elements of intrinsically motivated self-care. Examples include trying to understand what happened and how it can be avoided in the future, as well as seeking support from colleagues [52], [98], [101]. A universal solution is not to be expected here [102] (see figure 2 (Fig. 2)).

*Learning objective 5:* The graduate can identify the five levels of support and describe practical knowledge. Knowledge about the support options should be acquired first. Later, practical skills can be explained, including recognising when they are needed and the ability to request appropriate support.

*Learning objective 5.1:* The special significance of the ERNST model’s levels

*Learning objective 5.1.1:* The special significance of levels one and two. The graduate is aware of the significance of levels one and two of the ERNST model, can describe and apply them to themselves and third parties. 

*Depth of competence:* The first step is to impart knowledge about the existence of preventive measures at the individual and organisational levels. Elements of intrinsically motivated self-care for individuals and teams should also be identified. By the end of undergraduate study, students will have acquired the practical skills to apply preventive measures and self-care to increase resilience at the individual and organisational levels.

*Learning objective 5.1.2: *Special aspects of level three. Graduates can name the special position of level three of the ERNST model, describe its benefits and apply it to their practical skills for themselves and others.

*Depth of competence: *Knowledge of what a peer is and what secondary preventive effect peers have. Practical knowledge consists of being able to describe the skills of peers, and practical competence is demonstrated by the ability of the person concerned to request appropriate help from peers.

##### 4.3.3.2. Antonovsky’s sense of coherence

*Basic knowledge, learning objective 5.2: *An important key to dealing with stressful events is a person's resiliency [103], [104]. Based on Antonovsky’s sense of coherence, this is comprised of three subcomponents, which can be described as follows in relation to SVP:

1. Comprehensibility: recognition and understanding that a stressor is present;
2. Manageability: communication and support without fear of negative consequences;
3. Meaningfulness: awareness that professional stress can contribute to personal growth [6], [99].

*Learning objective 5.2:* The graduate can name the components of Antonovsky’s sense of coherence and describe ways to take action in terms of self-care. 

*Depth of competence:* Knowing these components can contribute to resilient coping with stressful situations. The focus here is initially on knowledge of the components, followed by the ability to communicate feelings of stress and seek help for oneself.

##### 4.3.3.3. Models of support

*Basic knowledge, learning objective 5.3: *Currently there is little empirical data on the effectiveness of SV programmes. A meta-analysis by Anger et al. showed that intervention programmes have a positive impact on the mental health of HCPs [31]. Outcome measures with a positive effect on SVs included stress levels, anxiety, depression, emotional exhaustion, and compassion fatigue. At the same time, a recent simulation study showed that SV programmes have a generally positive effect on employee well-being [31].

What all SV programmes have in common is that they provide information about SVP, establish a professional peer system while at the same time including higher levels of care. Most SVs received adequate care at levels two to three of the ERNST model [33], [70], [105], [106], [107].

Financially, it has been shown that peer support can achieve cost savings of 22,000 US dollars per case [108]. In Germany, a peer support programme for a hospital with a nursing staff of 1,000 saves 6.67 million euros per year [109].

*Learning objective 5.3: *The graduate can identify commonalities between best practice models of peer support, structured professional support, and structured clinical support and apply these to their own work.

For the depth of competence associated with learning objective 5.1, levels three to five of the ERNST model are particularly important in that structured peer support, psychosocial support from a specialist, and therapeutic counselling can be actively utilised. 

#### 4.3.4. Category IV: Contextualisation of SVP in the broader context of employee welfare

##### 4.3.4.1. Moral injury, overconfidence, overplacement, clinical tribalism 

*Basic knowledge, learning objective 6: T*he phenomenon of moral injury (MI) describes an acute violation of one’s own ethical framework. It develops simultaneously, subsequently or in connection with the moral dilemma and moral distress which describe the conflict between current actions and previous moral decisions [37], [42], [110], [111], [112]. Bushuven et al. demonstrated a link between SVP and MI, emphasising that the two can reinforce each other and that MI is particularly influenced by environmental factors [76], [113], [114]. For example, nurses with higher levels of MI display a stronger intention to leave their job permanently [115].

Three factors that can hinder the management of SVP are overconfidence, overplacement and clinical tribalism. Overconfidence describes the overestimation of one’s own abilities. Overplacement is the assumption that one is better than others. Clinical tribalism describes the overestimation of a group with which someone identifies [116], [117], [118]. In relation to SVP, these factors can lead to mistakes not being recognised as such. This attitude makes it difficult to admit stress, communicate, seek help and to see the point of doing so. 

*Learning objective 6: *The graduate can define the term “moral injury” and the associated phenomena of “overconfidence”, “overplacement” and “clinical tribalism” and explain how they act as barriers to getting support. 

*Depth of competence: *Knowing the above terms and being able to describe them in one's own words enables graduates to reflect on their actions and examine their own position with regard to barriers to support options. 

##### 4.3.4.2. Culture of safety, culture of uncertainty 

*Basic knowledge, learning objective 7: *Culture of safety: the surrounding system plays a major role when coping with stressful situations. Systemic support includes the provision of sufficient resources to respond to incidents. It also includes rules for case analysis, a culture in which mistakes are not punished per se and can be communicated openly, and a safety culture that prevents gossip, bullying and exclusion. At the individual level the safety system includes support for the SV [80], [93], [99], [100], [119], [120], [121], [122], [123], [124], [125], [126]. At the same time, institutional support for an SV is closely related to measures to improve the overall safety culture, increasing general well-being, reducing feelings of insecurity and decreasing symptoms of SVP and the intention to leave the workplace [112], [127], [128], [129], [130], [131]. A weakness of the system would be to provide insufficient support for an SV [132].

*Culture of uncertainty:* A culture in which mistakes are not dealt with openly can hinder learning from mistakes and thus compromise patient safety. When employees feel ashamed of their mistakes, trust in leadership and patient confidence in healthcare suffer. A culture of uncertainty creates barriers to offering and accepting support. There is a negative correlation between the quality of support provided by an SV and the psychological and professional consequences experienced [30], [92], [133], [134], [135], [136], [137], [138], [139], [140].

*Learning objective 7:* The graduate can provide examples of a culture of safety and a culture of uncertainty, contextualise them and apply them to their own situation.

*Depth of competence:* The graduate can identify instances of systemic support, reflect on them in relation to a culture of safety, know the options for supporting SVs, and communicate their own mistakes appropriately.

## 5. Discussion

In this paper we have derived seven learning objectives on SVP for medical students based on a comprehensive literature review. These objectives include a basic understanding of SVP, support tools for SVs, and the inclusion of SVP in the broader context of employee welfare. 

This presentation reflects the current state of research, although significant gaps remain. There are significant gaps in research with regard to:

1. the effectiveness of support programmes,
2. the effectiveness of preventive measures, including SVP training curricula,
3. the impact of factors such as gender, age, professional experience and cultural background on the experience of and coping with SVP,
4. the economic aspects of SVP.

Although various support programmes for SVs exist, there is little evidence-based research on the effectiveness of these programmes [99]. Although initial findings on factors influencing SVP are available [76], further research is needed, e.g., on the interaction of personality factors, moral injury, environmental factors and symptom burden. 

This paper can be seen as a contribution to closing a gap in research on preventive measures. A basic knowledge of SVP is required to recognise it at an early stage and thus prevent serious consequences [141]. Since students can already be affected, this knowledge should be anchored in the curriculum from the first year of study onwards.

In the current Catalogue of Competency-based Learning Objectives for Undergraduate Medicine (NKLM 2.0), section VIII.6-03.1 deals with the topic of self-reflection and self-awareness, and section VIII.6-03.2 deals with the topic of personal health and well-being. Some aspects of the above-mentioned learning objectives are already present, e.g., team-based error analysis, individual strategies for coping with and reducing stress (VIII.3-03.2; VIII.6-03.2.2). However, the NKLM 2.0 does not mention content or learning objectives that explicitly relate to SVP.

This contrasts with the fact that almost every HCP experiences SVP at least once in the course of their professional life [30], [142]. The current literature does not clearly indicate whether SVP has been explicitly incorporated into the medical curriculum. At the same time, the culture of safety in US hospitals has a much longer tradition than in Germany, at least since the book *To Err Is Human: Building a Safer Health System* formulated concrete demands for the development of a safety culture in healthcare organisations [34]. This culture of safety was the basis for many flagship projects related to SVP originating in the US [32], [33]. 

### Limitations 

Grant et al. identify 14 different review methods, with the approach of the present study best classified as qualitative evidence synthesis [26]. This method offers the advantage of combining research findings with field reports and practical observations, which enables a more comprehensive understanding of complex phenomena. However, it also presents challenges, as the methods are not clearly defined, which can lead to subjectivity in the interpretation and synthesis of findings. Also, this method carries the risk of possible sample bias in the selected literature.

The studies included here cover a wide variety of populations and work environments, and the methodologies used are very heterogeneous. Due to this diversity, a systematic review was neither feasible nor intended. 

## 6. Conclusion

The strength of this study lies in the fact that it is, to our knowledge, the first to define what medical students should know about SVP. Two further strengths are also significant:

1. The learning objectives formulated above provide a structured overview of SVP and can be implemented directly as a course.
2. The learning objectives focus on HCPs at the start of their careers, which maximises the preventive benefits.

*Teaching medical students about SVP early on in their studies is an essential part of self-care and a task for the healthcare system. This paper lays the groundwork for this, but further evaluation is needed to develop concrete teaching materials.*

## Authors’ ORCIDs

- Tobias Bexten: [0009-0002-5113-4589]
- Jens Christian Kubitz: [0000-0001-6634-5843]
- Anne Kamphausen: [0000-0002-2647-5202]
- Reinhard Strametz: [0000-0002-9920-8674]

## Competing interests

The authors declare that they have no competing interests. 

## Supplementary Material

Supplementary material

## Figures and Tables

**Table 1:**
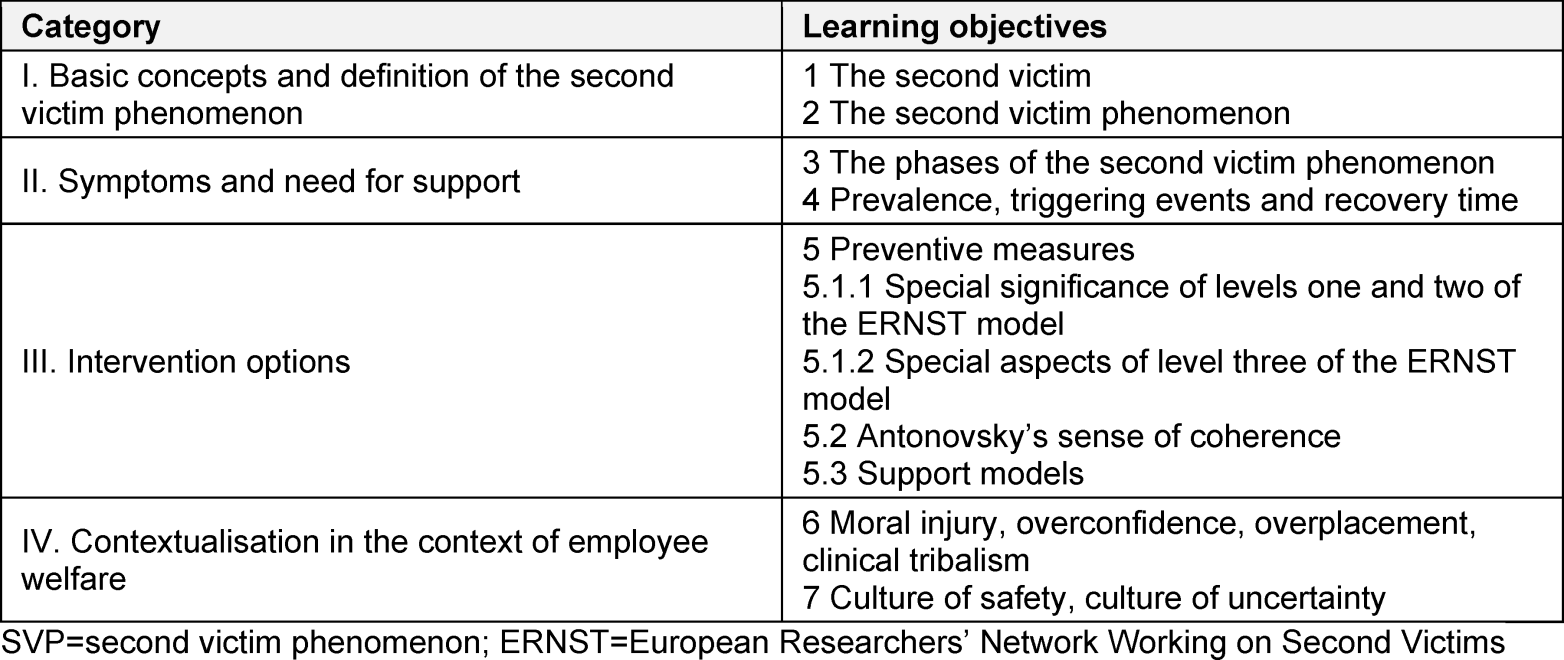
Categories and learning objectives

**Table 2:**
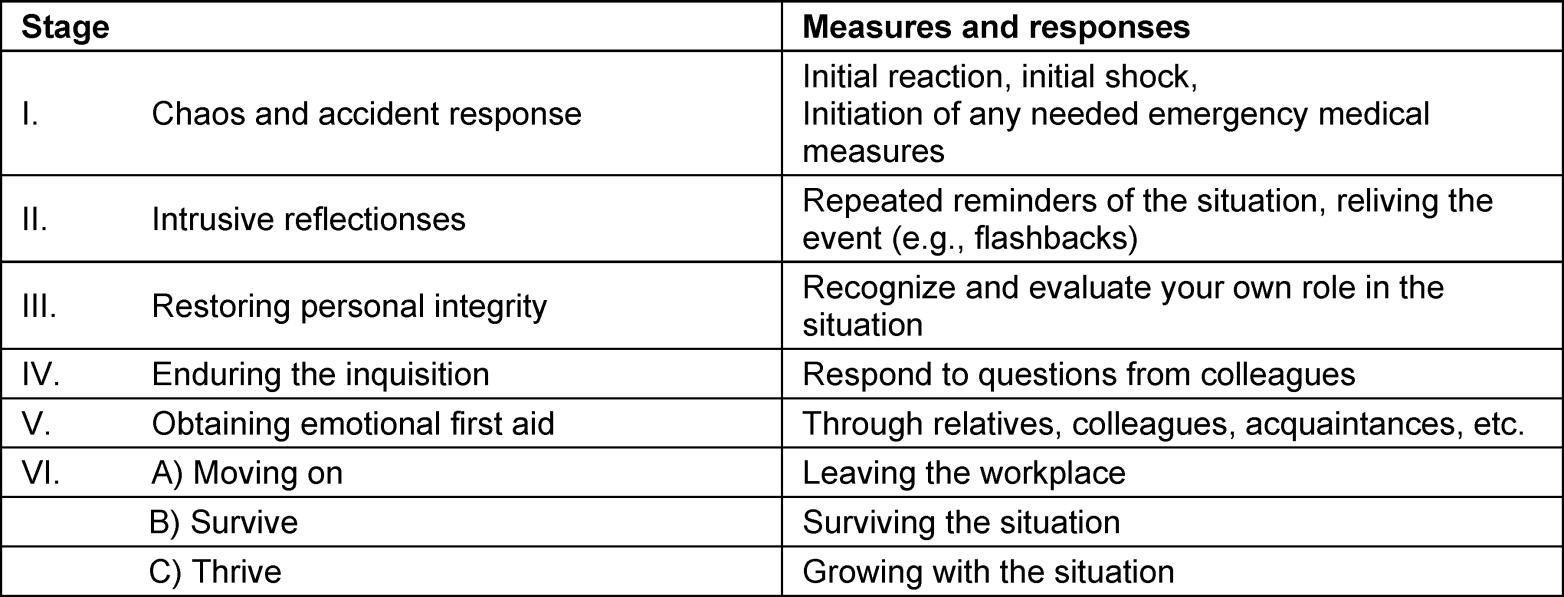
Stages of processing the second victim phenomenon This table describes six typical stages of reaction and processing after a stressful event according to S. Scott. *Inquisition was taken from the original work and refers not only to stressful questions from colleagues but also to questions such as: “Will I keep my job?”

**Table 3:**
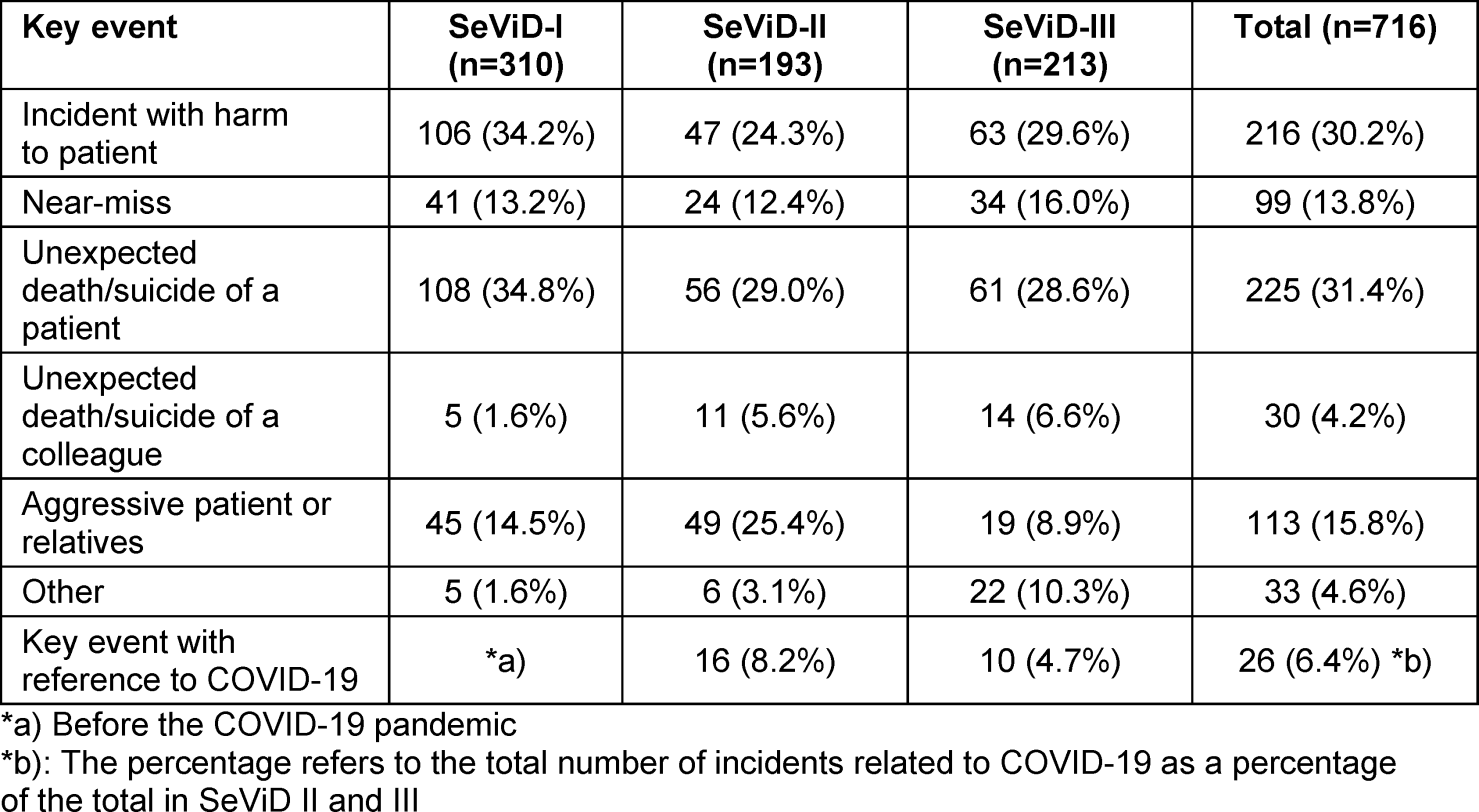
Frequency of key events experienced by SVs in Germany
