# Supplementary material for: The second victim phenomenon in medical education: Development of learning objectives based on the National Competency-based Catalogue of Learning Objectives for Undergraduate Medical Education (NKLM)
Source: GMS J Med Educ. 2025 Nov 17;42(5):Doc62. doi: 10.3205/zma001786 (PMC12661561; doi:10.3205/zma001786)
Supplement: Supplementary material [file JME-42-62-s-001.pdf]

Attachment 1: Supplementary material

Table S1: Search terms

Number	Queried on 7 April 2024
0	"Second Victim" OR "Second Victim Phenomenon"
and	
1	"definition"[All Fields]
2	"Prevalence" OR "Incidence"
3	"prevention"[All Fields]
4	"antonovsky aaron"[All Fields]
5	"moral injury"[All Fields]
6	culture of security
7	culture of insecurity
8	"events"[All Fields]
9	"reactions"[All Fields]
10	"experience"[All Fields]
11	"symptoms"[All Fields]
12	"phases"[All Fields]
13	"support"[All Fields]
14	"peer"[All Fields]
15	"support programme"[All Fields]
16	"just culture"[All Fields]
17	"no blame culture"[All Fields]

Figure S1: Flow chart illustrating study selection

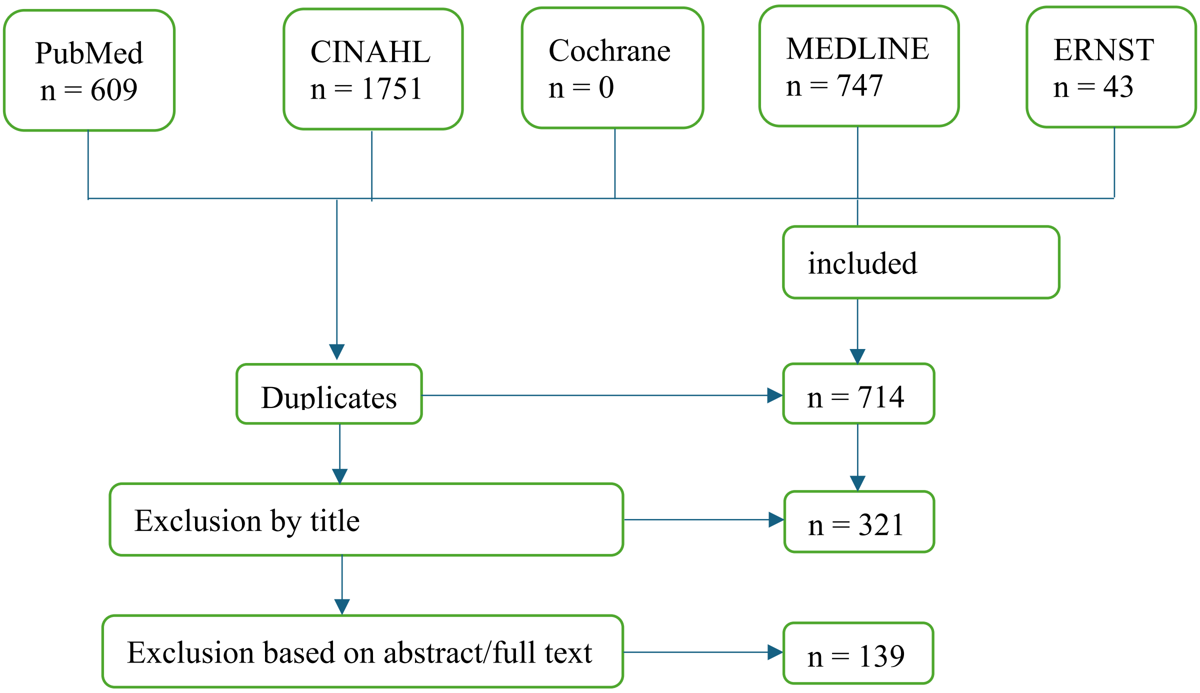

Attachment 1 to Bexten T, Kubitz JC, Kamphausen A, Strametz R. *The second victim phenomenon in medical education: Development of learning objectives based on the National Competency-based Catalogue of Learning Objectives for Undergraduate Medical Education (NKLM)*. GMS J Med Educ. 2025;42(5):Doc62. DOI: 10.3205/zma001786

Figure S2: Process of synthesis to develop learning objectives

Illustration of how the learning objectives were developed based on the ERNST website, accompanying literature (n=43), and structured database searches (CINAHL, PubMed, Medline). Using 17 search terms and four content categories, 139 publications were analysed and converted into seven learning objectives with the associated basic knowledge.

ERNST=European Researchers' Network Working on Second Victims.

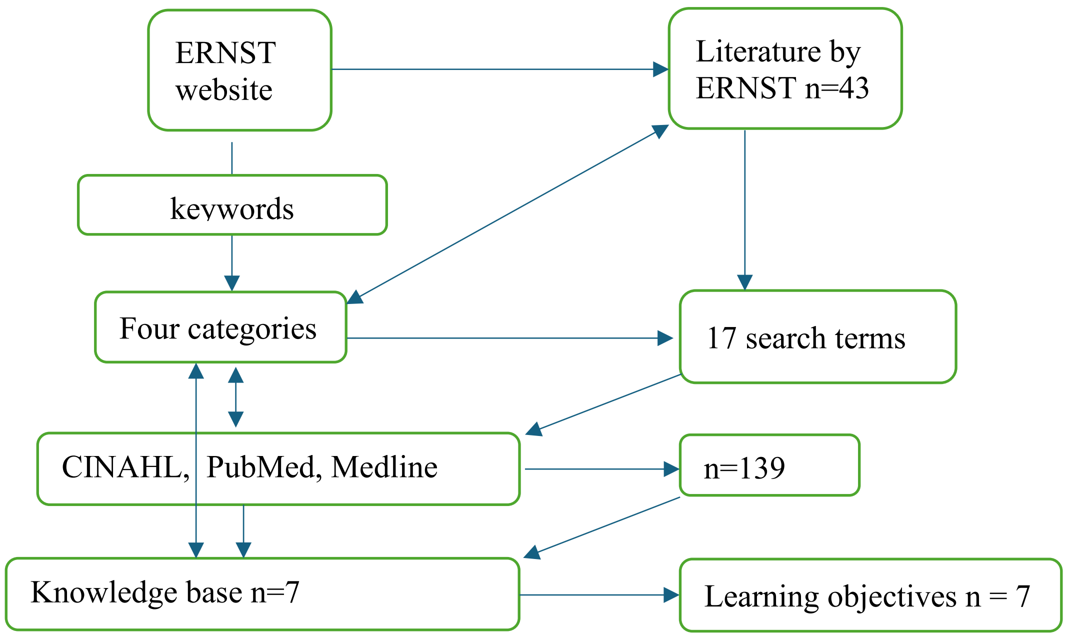

Attachment 1 to Bexten T, Kubitz JC, Kamphausen A, Strametz R. *The second victim phenomenon in medical education: Development of learning objectives based on the National Competency-based Catalogue of Learning Objectives for Undergraduate Medical Education (NKLM)*. GMS J Med Educ. 2025;42(5):Doc62. DOI: 10.3205/zma001786

Table S2: Overview of the learning objectives based on the taxonomy of the National Competency-based Learning Objectives for Medicine

	Competency/Learning objective	Sem. 1-4	Sem. 5-6	Sem. 7-10	PY	Defined by clarification and competence-based cross-references	Further defined by additional explanations	Type
	The graduate is familiar with and recognizes SVP.							Competency
	The graduate can explain SVP in detail, identify support options and apply strategies for self-care.							Sub-skill
1	The graduate can define the concept of SV and contextualize it using examples.	1	1	1	1	An SV is “any healthcare worker, directly or indirectly involved in an unanticipated adverse patient event, unintentional healthcare error, or patient injury and who becomes victimized in the sense that they are also affected is a second victim.”	The ability to define SV and describe it in their own words enables graduates to relate other aspects of SVP to themselves and their environment.	Learning objective
2	The graduate can describe the subjective experience of SVP. They have the ability to reflect on their own experience in relation to SVP and to deal with their own feelings.	1	1	1	2	A complex reaction manifested in psychological, cognitive, and/or physical symptoms, e.g., feelings of shock, fear, guilt, shame, sadness, or dissatisfaction with one's own actions.	Familiarity with the subjective experience of SVP and the ability to describe it in their own words enables graduates to reflect on their own experiences in relation to SVP. Graduates are mindful of and able to deal with symptoms of SVP.	Learning objective

3	The graduate can list the stages according to Scott et al. and explain the symptoms and actions/reactions based on them. The graduate can summarise stages one to five (non-linear) and can explain stage six according to the three possible resulting outcomes.	1	1	1	1	1. Chaos and accident response;<br>2. Intrusive reflections;<br>3. Restoring personal integrity;<br>4. Enduring the inquisition;<br>5. Obtaining emotional first aid;<br>6a. Moving on,<br>6b. Surviving,<br>6c. Thriving.	Familiarity with the subjective experience of SVP and the ability to describe it in their own words enables graduates to reflect on their experiences in relation to SVP. A direct call to action develops when further learning objectives are included.	Learning objective
4	The graduate can identify prevalence, triggering events, and recovery times for SVP.	1	1	1	1	Prevalence up to 60%; key events: harm to patients, near misses, unexpected death/suicide of patients, aggressive patients or relatives; recovery time: one third within a week, one third within a month, 15% within a year. 8% do not recover fully.	Knowing the triggering events and the fact that it is possible to recover fully from SVP is fundamental in terms of primary and secondary prevention. Only an SV who recognizes that they are affected will seek help when necessary.	Learning objective
5.1	The graduate can identify the individual levels of support options according to Seys et al. and describe practical knowledge.	1	1	2	2	Level 1: Prevention (individual and organizational)<br>Level 2: Self-care (individual and team)<br>Level 3: Peer support<br>Level 4: Structured professional support<br>Level 5: Structured clinical support	Cross-reference to 5.1.1 and 5.1.2: Practical knowledge in relation to levels four and five according to Seys et al., recognizing when these are needed and obtaining them for oneself, and requesting this level of support for others.	Learning objective

5.1.1	The graduate is aware of the special significance of levels one and two according to Seys et al., can describe them, and apply them in relation to themselves and third parties.	1	2	2	3b	Level 1: Prevention (individual and organizational)<br>Level 2: Self-care (individual and team)	Competence lies, first, in preventive measures at the individual level (error-minimizing safety measures, etc.) and at the organizational level (team building, communication training, etc.) and, second, in elements of intrinsically motivated self-care by individuals and teams to increase resilience before an event has occurred.	Learning objective
5.1.2	The graduate is aware of the special significance of level three (peer support) according to Seys et al., describe its benefits, and demonstrate the practical skills to use this tool for themselves and others.	1	1	2	3b	Level three: Peer support (colleagues trained in psychosocial emergency care)	This knowledge is demonstrated by describing what a peer is and what secondary preventive effect peer support has in relation to SVP (sem. 1-6). Practical knowledge lies in describing the peer's practical skills after an acute event (sem. 7-10). The practical skills involve requesting and obtaining appropriate help from peers when affected and requesting help for others (PY).	Learning objective

5.2	The graduate can name the components of Antonovsky's sense of coherence and describe ways to take action in terms of self-care.	1	1	2	3a	1. Comprehensibility<br>2. Manageability<br>3. Meaningfulness	Knowledge of the components of coherence enables individuals to perceive them more clearly in relation to their own resilience and to protect themselves from the negative consequences of SVP.	Learning objective
5.3	The graduate can name best practice models of peer support, structured professional support, and structured clinical support and implement them in their own work.	1	3a	3a	3b	1. Support from peers<br><br>2. Structured professional support<br><br>3. Structured clinical support	The ability to act lies in requesting and obtaining appropriate help when affected.	Learning objective
6	The graduate can define the terms moral injury, overconfidence, overplacement, and clinical tribalism, and describe their significance as barriers to support options.	1	1	2	3a	1. Moral injury<br><br>2. Overconfidence<br><br>3. Overplacement<br><br>4. Clinical tribalism	Graduates are able to reflect on their own actions and examine their own stance regarding barriers to support options.	Learning objective
7	The graduates can give examples of a culture of safety and a culture of uncertainty, contextualize them and apply them to themselves.	1	1	2	3b	Safety culture, fair handling of mistakes ("just culture")	Name and reflect on systemic supports (sem. 1-4; competence depth 1) what safety culture and just culture entail. Identify options to actively support an SV (sem. 5-10; competence depth 2). Apply safety culture and communicate one's own mistakes appropriately (sem. 5-10; competence depth 3a).	Learning objective
